# Departure from treatment protocol in published randomised controlled trials: a review

**DOI:** 10.1186/1745-6215-12-S1-A129

**Published:** 2011-12-13

**Authors:** Susanna Dodd, Ian White, Paula Williamson

**Affiliations:** 1 Department of Biostatistics, Institute of Translational Medicine, University of Liverpool, L69 3GS, UK; 2 MRC Biostatistics Unit, Institute of Public Health, Robinson Way, Cambridge CB2 0SR, UK

## Objectives

This review aimed to ascertain the extent to which the issue of departure from treatment protocol (DTP) is reported and addressed in published analyses of RCTs.

## Methods

One hundred publications of RCTs were randomly selected from those published in the BMJ, NEJM, JAMA and Lancet during 2008. Each trial report was reviewed to determine the extent and nature of reporting on DTP and whether statistical methods were used to deal with DTP for both benefit and harms analyses.

## Results

Even the most basic adherence information was not presented in some trials. Forty-two publications did not state how many patients actually initiated their randomised treatment. Information about treatment discontinuation can be vague and may not allow assessment of the number of patients who completed the treatment protocol.

Ninety-eight publications reported at least one form of DTP, including non-receipt of allocated treatment (39 trials), incomplete treatment in those who initiated allocated treatment (78), switching trial treatments (12), starting disallowed/non-trial treatment (4), starting open label treatment out of trial (7), contamination across groups (3) and other nonadherence to treatment dose or schedule (23). Treatment providers were reported to be nonadherent when delivering treatment in 12 trials.

More than half (50 (51%)) of the publications that reported DTP used some method to deal with it, but none were based on randomisation-preserving techniques. The most common method was based on per protocol (PP) analysis (46) (including one instance of using inverse probability of censoring weighting) often labelled as intention to treat (ITT) (18) or modified ITT (5), but missing data techniques (2) and as treated analyses (3) were also implemented. Less than 40% (26) of the 69 trials which presented harms analyses specifically defined harms analysis populations, and the majority of these definitions were based on actual treatment received (18). The majority (31) of the 43 trials that did not explicitly specify harms analysis population appeared to analysed harms outcomes using ITT. Twelve reports explicitly commented on the fact that DTP was likely to have influenced the observed treatment effect.

## Conclusions

DTP data presented in RCT publications, particularly related to treatment initiation and discontinuation, may be ambiguous or scant. Trialists often attempt to deal with DTP using variations of PP analysis, although they may be labelled as ITT; randomisation-preserving methods are not typically used. There appears to be confusion among trialists over the appropriate analysis population for harms outcomes in the presence of DTP.

